# Magic and memory: using conjuring to explore the effects of suggestion, social influence, and paranormal belief on eyewitness testimony for an ostensibly paranormal event

**DOI:** 10.3389/fpsyg.2014.01289

**Published:** 2014-11-13

**Authors:** Krissy Wilson, Christopher C. French

**Affiliations:** Anomalistic Psychology Research Unit, Department of Psychology, Goldsmiths, University of London, London, UK

**Keywords:** magic, memory, suggestion, social influence, paranormal belief

## Abstract

This study uses conjuring to investigate the effects of suggestion, social influence, and paranormal belief upon the accuracy of eyewitness testimony for an ostensibly paranormal event. Participants watched a video of an alleged psychic seemingly bending a metal key by the power of psychokinesis. Half the participants heard the fake psychic suggest that the key continued to bend after it had been put down on a table and half did not. Additionally, participants were exposed to either a negative social influence (a stooge co-witness reporting that the key did not continue to bend), no social influence, or a positive social influence (a stooge co-witness reporting that the key did continue to bend). Participants who were exposed to the verbal suggestion were significantly more likely to report that the key continued to bend. Additionally, more participants reported that the key continued to bend in the positive social influence condition compared to the other two social influence conditions. Finally, believers in the paranormal were more likely to report that the key continued to bend than non-believers.

## INTRODUCTION

For centuries, magicians have amazed audiences by apparently defying the laws of nature. Such effects were based upon a deep understanding of lay psychology but until recently, with few exceptions, academic psychologists have largely ignored the insights that the art of conjuring can provide to help understand the workings of the human mind. Thankfully, as this special issue demonstrates, this situation is changing. One of the ways in which the art of conjuring can be of service to psychological science is by providing means to study a range of psychological phenomena such as perception and memory. The experiment described in this report is one such example.

Over several decades, a great deal of research has demonstrated the unreliability of memory and in particular the fallibility of eyewitness testimony. Many kinds of memory distortion effects have been investigated including those due to the presentation of post-event misinformation (e.g., Eakin et al., 2003) and the use of misleading questions (e.g., Loftus, 1975) and even the formation of detailed false memories for complete episodes (e.g., Loftus and Pickrell, 1995). Recently, researchers have turned their attention to a particular form of misinformation effect known as memory conformity (e.g., Wright et al., 2000, 2009; Gabbert et al., 2003; Gabbert and Hope, 2013). Memory conformity is said to occur when an individual memory report of one person becomes more similar to another person’s following their discussion of an event.

In forensic contexts, similar accounts from multiple witnesses are likely to be accorded greater evidential weight than an uncorroborated account from a single witness. While such an assumption may be defensible, it fails to recognize that multiple witnesses to an unusual event such as a criminal act are very likely to discuss the event before any formal investigation takes place. Information exchanged during such discussions may potentially change or add to original recollections of what happened. Researchers have investigated how memory recall of pairs of eyewitnesses can become distorted if the two witnesses discuss what they believe to be the same event. Gabbert et al. (2003) had pairs of participants watch a video of a staged crime recorded in such a way that crucial details that were available on one recording were not available on the other and vice versa. For example, one version of the video showed a young woman actually steal some money whereas it was not clear in the other version if she had done so as it was filmed from a slightly different viewpoint. Dyads in one condition discussed the event prior to recall while participants in a control condition did not. It was found that a significant number of participants erroneously included items of information in their report of the event that had been acquired as a result of discussion with a co-witness. For example, many of the participants who had not actually seen the young woman take the money mistakenly reported that they had seen this act following the discussion. These findings were replicated by Wilson and French (2004).

In addition to reports of criminal acts, the accuracy of eyewitness testimony is also crucially important in assessing reports of ostensibly paranormal experiences (OPEs) and other anomalous events (French, 2003; French and Wilson, 2006). Anomalistic psychologists have argued that most reports of OPEs can be plausibly explained in non-paranormal, typically psychological, terms and specifically that cognitive biases known to characterize human thought may lead many people to believe they have experienced something paranormal when in fact they have not. Although a wide range of cognitive biases are potentially of relevance in this regard (French, 1992; French and Wilson, 2007; French and Stone, 2014), memory-related biases are amongst the most important. French (2003) and French and Wilson (2006) presented comprehensive reviews of investigations of the accuracy of eyewitness accounts of OPEs, concluding that anecdotal reports of such events should be treated with considerable caution in light of the proven unreliability of memory in such circumstances.

Wiseman and Morris (1995), for example, compared the recall of believers and disbelievers in the paranormal for the details of pre-recorded “pseudo-psychic” demonstrations, such as apparent metal-bending by psychokinesis. Believers tended to have poorer recall of the details of the demonstrations, particularly those details that would give some indication of the type of sleight of hand that was used to achieve the effects. Perhaps not surprisingly, the believers rated the demonstrations as being more “paranormal” than disbelievers.

Poor recall of the events taking place in séances was demonstrated as long ago as 1887 by Hodgson and Davey (1887), with similar findings being reported by Besterman (1932) and more recently by Wiseman et al. (1995). In all such studies, all of the effects were achieved by the use of trickery based upon accounts from fake mediums. However, the accounts provided by eyewitnesses were often so inaccurate that, taken at face value, they would defy rational explanation. Once again, important details of the events that would have provided clues as to how the effects actually had been achieved were simply not recalled accurately.

Wiseman et al. (2003) examined the effects of suggestion during fake séances. In their first experiment, around a third of the witnesses erroneously reported that a stationary table had moved during the séance following a suggestion from the fake medium to this effect. Believers in the paranormal were more likely to misreport such movement than disbelievers. Believers were shown to be more susceptible to suggestion than disbelievers in a second set of fake séances too, but only when the suggestion was congruent with their belief in the paranormal. For example, if the fake medium suggested that an object had not moved when in fact it had (by trickery), believers were no more likely to accept the suggestion than disbelievers. Overall, around one-fifth of the participants believed they had witnessed genuine paranormal phenomena. As Wiseman et al. (2003) point out, it is unclear whether the verbal suggestion directly affected the participants’ perception of the event, their memory of the event, or both. It is even possible that neither perception nor memory was affected and that the results were due to demand characteristics, but the end result is the same: a large minority of the participants were willing to report that stationary objects had moved and that they had witnessed genuinely paranormal events.

Wiseman and Greening (2005) explored the power of verbal suggestion in another ostensibly paranormal context. In two experiments, participants were shown a videotape of an alleged psychic bending a key using apparent psychokinetic ability but in fact using sleight of hand techniques. Participants in one condition heard the psychic suggest that the key continued to bend after being put down on a table, whilst those in a second condition did not. The findings revealed that those in the suggestion condition were significantly more likely to report that the key had indeed continued to bend (even though it had not). The size of this effect was considerable, with around 40% of the participants in the suggestion condition reporting that the key continued to bend compared to virtually no one in the no-suggestion condition. Somewhat surprisingly, in the light of findings from the séance studies, no differences were found between believers in the paranormal and disbelievers in either experiment. In the second experiment (but not the first), those who erroneously reported that the key continued to bend were more confident regarding their recall than those who correctly reported that it did not. Interestingly, they were also significantly less likely to remember hearing the actual verbal suggestion provided by the fake psychic.

Recent studies have applied memory conformity paradigms to the study of OPEs on the assumption that witnesses of such events are very likely to discuss what they saw and one person’s report may influence the memory of other witnesses. Thus, if one witness to a séance, for example, was initially unsure whether a particular object had or had not moved during the séance, confident testimony from a fellow witness that it did may be sufficient to alter the first witness’s report of the event. Wilson (2006) used the same basic paradigm as that used by Gabbert et al. (2003) but with videotapes of two 2.5-min clips of a pseudo-psychic demonstration of apparently psychokinetic ability. Both clips contained essentially the same sequence of events but each included one important piece of information missing in the other clip, information that gave an indication of how the effect was achieved. In the first clip, for example, a fork used in a fork-bending demonstration is clearly handled by the alleged psychic and in the second, the fork clearly goes out of view. As with the previous study, the focus of interest was the degree to which the participants’ recall was distorted as a result of discussion with a co-witness. Once again it was found that a substantial majority of participants included crucial items of information about the event they witnessed that were most likely to have been acquired as a result of such discussions. This study therefore demonstrated that, as predicted, memory conformity effects do in fact occur in apparently paranormal contexts.

This general line of research is important for two main reasons. The first is that it provides an explanation of reports of various OPEs in terms of known psychological factors. Opinion polls repeatedly show that a large proportion of the population believes in the paranormal and a sizeable minority claims to have had direct personal experience of paranormal events. But, with a few notable exceptions, psychology has had little to say about the origins of such beliefs and experiences until fairly recently. We strongly believe, in line with other researchers within anomalistic psychology, that it is not enough simply to speculate upon the various psychological factors that may underlie reports of OPEs. It is important to support such accounts with empirical evidence and the current research is aimed at doing precisely this with respect to the factors of verbal suggestion and memory conformity.

The second main reason for carrying out such research is for what it can tell us about memory more generally. For example, most previous research into the reliability of eyewitness testimony has been carried out in a forensic context, often involving the use of staged crimes and so on. Apart from the obvious importance in terms of generalisability of studying such effects in a different context, investigating the reliability of reports of OPEs under controlled conditions offers an ideal opportunity to demonstrate the effects of pre-existing beliefs upon perception and memory. By their very nature, OPEs are often inherently ambiguous and it is precisely in such circumstances that we would expect top-down influences upon perception (French, 2001) and memory (French, 2003; French and Wilson, 2006) to be most pronounced.

The choice of belief in the paranormal as a means of exploring the influence of top-down processes on cognition is particularly appropriate for several reasons. In addition to the inherent ambiguity of most OPEs, (i) paranormal belief is prevalent in all societies, (ii) belief in the paranormal is very important in many people’s lives and such beliefs have strong emotional ties (e.g., the belief in life after death) and (iii) paranormal beliefs often form part of a larger set of beliefs and attitudes toward such things as religion, science and indeed mankind’s place in the universe. Furthermore, standard scales are available to measure the level of paranormal belief making it an ideal choice for this type of investigation.

The current study aimed to replicate and extend previous studies of verbal suggestion and memory conformity by systematically manipulating both the presence or absence of a verbal suggestion as well as the type of social influence exerted by a co-witness. Replication of such effects is crucially important in light of current concerns regarding poor replicability within psychology (see, e.g., Pashler and Wagenmakers, 2012; Ritchie et al., 2012). The study is based upon Wiseman and Greening’s (2005) demonstration of the power of verbal suggestion in the context of an alleged demonstration of psychokinetic metal-bending. Using the same video clip as that used in the original study, participants viewed a fake psychic apparently using psychokinesis to bend a key. After the psychic had put the bent key down, half of the participants heard the fake psychic suggest that the key continued to bend while the other half did not hear the suggestion. It was hypothesized, in line with the findings of the original study, that those in the suggestion condition would be more inclined to report that the key continued to bend in comparison to participants in the no-suggestion condition.

Furthermore, each participant was also exposed to one of three types of social influence from a co-witness. One-third of the participants were exposed to a “negative” social influence, insofar as the co-witness, during a post-event discussion, reported that the key did not continue to bend. Another third of the participants were not exposed to any social influence, as they did not discuss the demonstration at all. The final third of the participants were exposed to a “positive” social influence, in that the co-witness, during the post-event discussion, reported that the key did indeed continue to bend. The co-witness in the negative and positive social influence conditions was in fact a stooge. It was hypothesized, based upon previous memory conformity research, that the genuine participants in the positive social influence condition would be more inclined to report that the key continued to bend than those in the no social influence condition, whereas those in the negative social influence condition would be relatively less inclined.

Even though Wiseman and Greening (2005) did not find any difference between believers in the paranormal and disbelievers in terms of tendency to report that the key continued to bend, it was hypothesized in the current study that the former group may show this tendency more strongly on the basis of previous research including studies of susceptibility to suggestion in the séance room.

A number of individual difference measures have been shown to be correlated with both paranormal belief and tendency to report anomalous experiences on the one hand and susceptibility to various kinds of memory distortion on the other, including susceptibility to false memories (French, 2003; French and Wilson, 2006). This suggests that at least some reports of anomalous events may be based upon false memories. Dissociativity, for example, has been shown in a number of studies to correlate with paranormal belief (e.g., Irwin, 1994; Pekala et al., 1995; Wolfradt, 1997; Makasovski and Irwin, 1999; Rattet and Bursik, 2001) and the tendency to report a wide range of paranormal and anomalous experiences (e.g., Richards, 1991; Ross et al., 1991; Ross and Joshi, 1992; Pekala et al., 1995), as well susceptibility to false memories (e.g., Eisen and Carlson, 1998; Hyman and Billings, 1998; Winograd et al., 1998; Heaps and Nash, 1999; Ost et al., 2005; Wilson and French, 2006). One possible explanation for the link between dissociativity and susceptibility to false memories is that, by definition, high scorers on measures of dissociativity experience more disruptions in the integration of thoughts, awareness, and memory. Such individuals may therefore be more prone to accepting externally presented information as autobiographical memories.

The relationship between dissociativity and suggestibility is complex, but several studies have reported a significant correlation using a variety of measures of suggestibility (see Eisen and Lynn, 2001; Eisen et al., 2002). Therefore, given the known correlation between paranormal belief and dissociativity, a measure of dissociativity (the Dissociative Experiences Scale, DES) was administered in order to allow the assessment of possible effects of dissociativity upon the dependent variables in this study.

Compliance (or eagerness to please) has also been shown to be related to susceptibility to false memories (e.g., Ost et al., 2002, 2005). It might be expected that in the current experiment, where, depending upon the allocated condition, participants may be exposed to social influence in terms of the initial verbal suggestion from the fake psychic and/or the comments of the stooge, level of compliance would be related to the degree to which participants report that the key continued to bend. Therefore, the current study also measured compliance, using Snyder’s (1974) Self-Monitoring Scale (SMS).

The current study complied with the ethical guidelines of the British Psychological Society and ethical approval to conduct the study was granted by the Ethical Committee of the Department of Psychology, Goldsmiths College, University of London.

## MATERIALS AND METHODS

### PARTICIPANTS

One hundred and eighty undergraduates and college employees from Goldsmiths College, University of London, took part in the study. Participants were 144 females and 36 males with a mean age of 24.41 years (SD = 3.45) and an age range of 18–57 years. All participants responded to a poster advertising for involvement in an experiment where participants would be asked to judge the paranormal abilities of a professed psychic. Participants received either course credit or £5 for their involvement.

### DESIGN

This study generally employed a 2 × 3 × 2 factorial design with Verbal Suggestion (suggestion vs. no suggestion), Social Influence (negative social influence vs. no social influence vs. positive social influence), and Belief Group (believers vs. non-believers), as between-group factors. The primary dependent variable was scores on item 3 of a Fixed Response Questionnaire (FRQ3) asking participants to rate their degree of agreement with the statement “After the key was placed on the table, it continued to bend” (see below for details).

### MATERIALS

#### Videotape

The videotape used in the study was supplied by Richard Wiseman and is the same videotape as that used in Wiseman and Greening’s (2005) experiments. Two versions of the tape were used. In the *suggestion* version of the tape the film consists of a 2-min clip of an interviewer and “psychic” sat at a table with several objects such as cutlery and keys in front of them. The interviewer briefly introduces the psychic and invites him to perform a demonstration of his powers using any of the objects of his choice. The psychic then picks up a key and appears to use his psychokinetic powers to bend the key to a 25° angle, in fact achieving this effect by the use of sleight of hand. He then places the key back on the table and suggests that the key is in fact still bending, even though it is not. The *no verbal suggestion* version of the tape is identical to the *suggestion* version but part of the soundtrack was removed so that participants did not hear the verbal suggestion. The fake psychic used in the demonstration was in fact a magician who had worked professionally for many years using sleight of hand techniques.

#### Questionnaires

***Fixed Response Questionnaire.*** This is the 4-item questionnaire used by Wiseman and Greening (2005) and consists of statements concerning the film. Two of the statements are filler items, e.g., “The interviewer touched the items on the table.” Responses to the third item (FRQ3) were used as the main dependent variable in the study: “After the key was placed on the table, it continued to bend.” The fourth item on the questionnaire asked participants to what extent they considered the demonstration involved paranormal forces. For each item, participants were asked to provide their response on a 7-point scale from 1 (*Definitely No*) to 7 (*Definitely Yes*). Participants were also asked to rate their confidence in their answers on a similar scale from 1 (*not at all confident*) to 7 (*very confident*).

***Forced-choice version of the Australian Sheep-Goat Scale.*** This is a widely used scale that consists of 18 statements relating to the three core concepts of parapsychology: extrasensory perception, psychokinesis, and life after death. The statements refer to belief in and alleged experience of the paranormal and respondents are awarded no points for a “false” response, one point for a “don’t know” response, and two points for a “true” response (allowing for a maximum score of 36). Note that this scale was preferred to the unstandardized Belief in the Paranormal Questionnaire used by Wiseman and Greening (2005) because it has known validity and reliability (e.g., Thalbourne and Delin, 1993; Thalbourne, 1995, 2010) and it allowed comparison with other research in this area (e.g., Wilson and French, 2006). Scores on this scale were used to allocate participants to belief groups.

***Dissociative Experiences Scale.*** This scale, designed and developed by Bernstein and Putnam (1986), consists of a 28-item self-report questionnaire. A typical example would be: “Some people have the experience of finding new things among their belongings that they do not remember buying.” Respondents are asked to circle a box to indicate what percentage of the time this event happens to them, ranging from 0 to 100% at 10% intervals. Each item is awarded a score between 0 and 100 and the mean score is then calculated across the 28 items. The scale has been shown to have good psychometric properties (Dubester and Braun, 1995) and internal consistency (Norton et al., 1990).

***Self-Monitoring Scale of Expressive Behaviour.*** This scale, developed by Snyder (1974), is a 25-item true–false questionnaire consisting of items such as “When I am uncertain how to act in a social situation, I look to the behavior of others for cues” and “My behavior is usually an expression of my true inner feelings, attitudes, and beliefs.” The score on this scale indicates the extent to which respondents rely on cues from others in deciding how to behave in social situations as opposed to relying upon personal values. One point is awarded for every response in line with such tendencies.

### PROCEDURE

All participants were told that they were to judge the paranormal powers of a professed “psychic” who had claimed to the Psychology Department that he could demonstrate psychokinetic ability. Participants were allocated to one of the six experimental conditions produced by crossing the two factors of Verbal Suggestion (suggestion vs. no suggestion) and Social Influence (negative social influence vs. no social influence vs. positive social influence). To maintain comparability across all experimental conditions, all participants were tested in pairs. In the *positive* and *negative social influence* conditions, one of the apparent participants was in fact a stooge playing the part of a co-witness, whereas in the *no social influence* condition both participants were genuine. Participants in the *suggestion* condition watched the video with an audible commentary throughout, whereas those in the *no suggestion* condition were not presented with the verbal suggestion from the fake psychic.

In the *no social influence* conditions, both participants watched the video and were then asked to complete the questionnaires. In the *positive* and *negative social influence conditions*, the stooge and the participant arrived at the testing room at the same time. Both watched the video but were then told to discuss the details of the film together. In order to facilitate this discussion, the stooge and the real participant were asked to complete a short questionnaire consisting of four questions relating to the film. These included three filler questions, e.g., “What was the psychic wearing?” and the crucial question, i.e., “Did the key continue to bend after it had been placed on the table?” Participants were told to complete this brief questionnaire together. The stooge was instructed to speak first and to lead the discussion, either maintaining that the key had continued to bend and that paranormal forces had been at work (in the *positive* condition) or that the key had not continued to bend and that no paranormal forces were involved (in the *negative* condition). After the discussion the participants independently completed the other questionnaires.

At the end of the experiment, all participants were debriefed fully. Participants in the *positive* and *negative social influence* conditions were asked if at any time they had suspected that their fellow co-witness was a confederate of the researcher. However, no participants reported that they had been suspicious of the stooge. To maintain continuity the same stooge took part in all the trials.

## RESULTS

Participants were first classified into Belief Groups on the basis of a median split of Australian Sheep-Goat Scale (ASGS) scores, with those scoring more than 10 classified as believers and the rest as disbelievers in the paranormal. It is common practice in studies comparing high and low paranormal belief groups on performance measures to divide the groups using a median split on the belief measure as done by Wiseman and Greening (2005) and in the current study. Although this approach runs the potential risk of failing to detect real effects because information is lost by converting a continuous variable to a binary variable (MacCallum et al., 2002), one can be certain that any effects identified with this approach would also be found using alternative methods such as multiple regression. Indeed, results from the current study were also analyzed using multiple regression techniques and the pattern of results found was identical to that reported below. However, it was felt that the effects found were described more clearly using the results of ANOVAs.

In order to check that unintended sampling bias had not been introduced by splitting our sample in this way, three 2 × 2 × 3 ANOVAs were carried out on the scores from the ASGS, DES, and SMS, respectively, each with Belief Group, Verbal Suggestion, and Social Influence as between-group factors. As would be expected given the method of allocation to belief groups, ASGS scores were significantly higher for believers (mean = 18.25, SD = 4.80) than disbelievers [mean = 3.78, SD = 3.05; *F*(1,168) = 562.26, *p* < 0.001]. Also, as expected given the known correlation between paranormal belief and dissociativity, DES scores were significantly higher for believers (mean = 36.99, SD = 15.44) than for disbelievers [mean = 28.05, SD = 16.12; *F*(1,168) = 12.81, *p* < 0.001]. Interestingly, SMS scores were also significantly higher for believers (mean = 12.51, SD = 3.65) than disbelievers [mean = 10.87, SD = 4.08; *F*(1,168) = 6.04, *p* = 0.015]. No other main effects or interactions from any of the three ANOVAs were statistically significant. ASGS scores correlated significantly with both DES scores (*r* = 0.278, *p* < 0.001) and SMS scores (*r* = 0.180, *p* = 0.016) across the sample as a whole.

Next, responses to FRQ3 were analyzed using a 2 × 2 × 3 ANOVA with the same factors as those used in the previous analysis. This analysis revealed a significant main effect of Verbal Suggestion, with participants who heard the suggestion giving higher ratings on FRQ3 (mean = 3.92, SD = 2.02) than those who did not [mean = 2.56, SD = 1.74; *F*(1,168) = 32.40, *p* < 0.001]. A main effect of Social Influence was also found [*F*(2,168) = 22.01, *p* < 0.001]. Using Bonferroni-adjusted t-tests, it was shown that positive social influence produced higher ratings on FRQ3 (mean = 4.43, SD = 1.96) than either negative social influence [mean = 2.50, SD = 1.54; *t*(118) = 6.02, *p* < 0.001] or no social influence [mean = 2.78, SD = 1.94; *t*(118) = 4.63, *p* < 0.001]. However, the two latter conditions did not produce significantly different ratings [t(118) = 0.89, n.s.]. Finally, believers in the paranormal gave significantly higher ratings on FRQ3 (mean = 3.75, SD = 1.97) than disbelievers [mean = 2.75, SD = 1.93; *F*(1,168) = 9.94, *p* = 0.002]. No significant interactions were found.

In light of the significant differences between belief groups on scores for the DES and SMS, these variables were entered as covariates in the main analysis of responses to FRQ3 in order to ascertain whether any differences found between belief groups could be accounted for in terms of differences between the groups on these variables. Therefore responses to FRQ3 were analyzed using a 2 × 2 × 3 ANOVA with the same factors as those used in the previous analysis, but with the inclusion of DES and SMS scores as covariates. This analysis revealed a significant main effect of Verbal Suggestion, with participants who heard the suggestion giving higher ratings on FRQ3 (mean = 3.92, SD = 2.02) than those who did not [mean = 2.56, SD = 1.74; *F*(1,179) = 32.05, *p* < 0.001].

A main effect of Social Influence was also found [*F*(2,179) = 21.06, *p* < 0.001]. Using Bonferroni-adjusted t-tests, it was shown that positive social influence produced higher ratings on FRQ3 (mean = 4.43, SD = 1.96) than either negative social influence [mean = 2.50, SD = 1.54; *t*(118) = 6.02, *p* < 0.001] or no social influence [mean = 2.78, SD = 1.94; *t*(118) = 4.63, *p* < 0.001]. However, the two latter conditions did not produce significantly different ratings [t(118) = 0.89, n.s.].

Finally, even with DES and SMS scores entered as covariates, believers in the paranormal gave significantly higher ratings on FRQ3 (mean = 3.75, SD = 1.97) than disbelievers [mean = 2.75, SD = 1.93; *F*(1,179) = 7.89, *p* = 0.006]. DES and SMS scores were not significantly related to responses on the FRQ3 in this analysis. Once again, no significant interactions were found.

Following Wiseman and Greening (2005), participants were then allocated to two groups depending upon their responses to the FRQ3. Those who responded with either a 5, 6, or 7 were allocated to the *key continued to bend* group. The rest were allocated to the *key did not continue to bend* group. The numbers and percentages in each group across experimental conditions are presented in Table 1. Chi-square analyses between group and suggestion within each social influence condition revealed a highly significant effect (χ^2^ = 12.0, df = 1, *p* = 0.001), in the no social influence condition (thus replicating Wiseman and Greening, 2005) with 10 participants (33.3%) reporting that the key continued to bend if given the verbal suggestion compared to none in the no-suggestion condition. Neither of the other chi-square analyses was significant. It is worth noting that the percentage of participants in the suggestion condition reporting that the key continued to bend was decreased to 23.3% in the negative social influence condition and almost doubled to 60% in the positive social influence condition (χ^2^ = 6.9, df = 1, *p* = 0.009).

**Table 1:** **Numbers and percentages of participants in the *key continued to bend* and the *key did not continue to bend* groups across experimental conditions.**

	Key continued to bend group	Key did not continue to bend group
Negative social influence		
Suggestion	7 (23.3%)	23 (76.7%)
No suggestion	2 (6.7%)	28 (93.3%)
No social influence		
Suggestion	10 (33.3%)	20 (67.7%)
No suggestion	0 (0%)	30 (100%)
Positive social influence		
Suggestion	18 (60%)	12 (40%)
No suggestion	12 (40%)	18 (60%)

Wiseman and Greening (2005, Experiment 2) found that those reporting that the key continued to bend were more confident about the accuracy of their report than those who reported that it did not (although this result was not found in their first experiment). Responses to item 3b of the FRQ in the current study, indicating confidence in the accuracy of participants’ reports on FRQ3, are presented in Table 2. These data were subjected to a 2 × 3 ANOVA with Bend Group (did continue to bend vs. did not continue to bend) and Social Influence Group as between-groups factors. No significant main effects were found but a highly significant interaction was revealed [*F*(2,179) = 5.25, *p* = 0.006]. Further exploration of this interaction, using three Bonferroni-adjusted t-tests, revealed only one significant effect: participants in the positive social influence condition who reported that the key continued to bend were far more confident in their ratings than those who reported that the key did not continue to bend [*t*(58) = 3.51, *p* = 0.001]. The same general trend was evident for those in the no social influence group although the opposite trend was evident for those in the negative social influence group, i.e., in the latter condition, those reporting that the key continued to bend were relatively *less* confident than those who reported that it did not. A 2 × 2 ANOVA using the same factors as those in the previous analysis but excluding the positive social influence group revealed that the interaction between Bend Group and Social Influence was still significant [*F*(1,119) = 5.13, *p* = 0.025].

**Table 2:** **Mean confidence ratings (SDs in parentheses) given to item FRQ3 by participants in the *key continued to bend* and the *key did not continue to bend* groups across experimental conditions.**

	Key continued to bend group	Key did not continue to bend group
Negative social influence	4.56 (1.59), *N* = 9	5.53 (1.84), *N* = 51
No social influence	6.20 (1.48), *N* = 10	5.24 (1.62), *N* = 50
Positive social influence	6.07 (1.02), *N* = 30	4.73 (1.82), *N* = 30

Wiseman and Greening (2005) did not report any analyses of responses from the fourth item on the FRQ (FRQ4), dealing with the degree to which participants believed the demonstration, including the initial key bending by sleight of hand, involved paranormal forces. FRQ4 data from the present study were subjected to a 2 × 2 × 3 ANOVA with Belief Group, Verbal Suggestion, and Social Influence as between-group factors. Not surprisingly, believers in the paranormal gave higher ratings (mean = 3.32, SD = 1.64) than disbelievers [mean = 1.87, SD = 1.18; *F*(1,179) = 44.38, *p* < 0.001]. Perhaps more surprisingly, higher ratings were given by participants exposed to the verbal suggestion (mean = 2.90, SD = 1.73) than those who were not so exposed [mean = 2.26, SD = 1.39; *F*(1,179) = 9.57, *p* = 0.002]. The generally low levels of ratings of paranormality should, however, be noted.

Participants were then allocated to groups on the basis of whether they did or did not believe the demonstration involved paranormal forces. Those scoring either 5, 6, or 7 on FRQ4 were allocated to the *demonstration was paranormal group* and the rest were allocated to the *demonstration was not paranormal group.* The numbers and percentages in each group across the experiment as a whole are presented in Table 3. Across the experiment as a whole, 49 out of 180 participants (27.2%) reported that the key continued to bend and 23 (12.8%) believed they had witnessed something paranormal in the demonstration as a whole. Of the 88 believers, 33 (37.5%) reported that the key continued to bend and 20 (22.7%) believed they had witnessed paranormal forces in action. Note that this implies that many of the believers who reported that the key carried on bending did not believe that this particular demonstration involved genuine paranormal forces, presumably believing instead that it was based upon some form of trickery. Of the 92 disbelievers, only 16 (17.4%) reported that the key continued to bend and only 3 (3.2%) of those classified as disbelievers reported that they had witnessed paranormal forces in action. Presumably, this tiny percentage of “disbelievers” who believed they had witnessed a genuine paranormal event had been so classified because they did not believe in life after death and/or ESP even though, evidently, they did believe in psychokinesis. It is interesting to compare the interpretation of the demonstration between the belief groups for those participants who reported that the key did continue to bend. Of 33 believers who reported that the key continued to bend, 13 (39.4%) reported that the demonstration was paranormal. Of 16 disbelievers who reported that the key continued to bend, only one (6.2%) reported that the demonstration was paranormal (χ^2^ = 5.8, df = 1, *p* = 0.016). Clearly, disbelievers were much more likely to opt for a non-paranormal explanation even if they believed they had seen the key carry on bending.

**Table 3:** **Number and percentages of participants in the *demonstration was paranormal* and the *demonstration was not paranormal* groups across the experiment as a whole, broken down by Belief Group and Bend Group.**

	Demonstration was paranormal	Demonstration was not paranormal
Believers		
Key continued to bend	13 (39.4%)	20 (60.6%)
Key did not continue to bend	7 (12.7%)	48 (87.3%)
Disbelievers		
Key continued to bend	1 (6.2%)	15 (93.8%)
Key did not continue to bend	2 (2.6%)	74 (97.4%)

## DISCUSSION

The results of this experiment largely confirm the basic finding of Wiseman and Greening’s (2005) experiments; that is, in this context, a relatively mild verbal suggestion from a fake psychic that a bent key continued to bend after it had been placed upon a table was sufficient to lead a substantial number of witnesses to erroneously report that the key had indeed done just that. In the no social influence condition in the current experiment, the condition most similar to that used by Wiseman and Greening (2005), one-third of the participants reported continued bending, compared to 39.13% in their Experiment 1 and 36.54% in their Experiment 2. Also in line with Wiseman and Greening’s (2005) findings, no participants in the *no suggestion* and *no social influence* condition reported continued bending.

The current study extended the findings of the original experiments by incorporating an additional social influence component into the design. When a stooge co-witness insisted that the key continued to bend, 60% of the participants agreed. When the stooge co-witness insisted that the key did not continue to bend, the percentage who reported that it did was substantially reduced, but even then 23.3% reported that it did. This is a powerful demonstration that it is not only what witnesses to an ostensibly paranormal event believe that they have actually perceived at the time that determines their subsequent reports but that such reports will also be influenced by discussion with co-witnesses in line with findings from memory conformity research.

We also found one result that was not in line with the findings of the original experiments by Wiseman and Greening (2005). In the current study, believers in the paranormal were found to be more likely to report that the key continued to bend compared to disbelievers. Wiseman and Greening (2005) considered two possible explanations for their failure to find any difference between belief groups. First, they considered the possibility that previous studies reporting an association between paranormal belief and suggestibility might be mistaken, possibly reflecting a “file-drawer” effect in which a few studies finding a spuriously significant relationship between these two variables had been published but that they should be considered in the wider context of a possibly much larger number of studies that had tried and failed to find such an effect and had therefore never been submitted for publication. Second, they suggested that paranormal belief may correlate with certain kinds of suggestibility but not the form of suggestibility involved in their key-bending experiments. The current findings would argue against both of these suggestions. It appears that the type of suggestibility involved in both the original experiments and the current study is indeed correlated with paranormal belief. The most likely explanation for the discrepancy between Wiseman and Greening’s (2005) findings in this regard and the findings of the current study is our decision to use the ASGS as a measure of paranormal belief. Furthermore, the belief-related effects found were not explicable in terms of differences between the belief groups on the DES and SMS measures.

Wiseman et al. (2003), in the context of discussing the effects of suggestion on eyewitness reports in the séance room, acknowledge that it is often difficult to determine whether verbal suggestion directly affects the perception of the event, memory for the event, or both. It is even possible that neither is affected and that the results are due to demand characteristics. Thus, it is possible that the verbal suggestions during the séance directly influence the perception of the witnesses in such a way that those witnesses who are exposed to such suggestions actually perceive stationary objects to be moving in real time. Alternatively, it is possible that the witnesses did not actually perceive the stationary objects to be moving at the time but that their memories of the event were affected by the verbal suggestions when, 2 weeks later, they received a questionnaire asking them to recall details of the séance. By that time, their memory for the séance would be beginning to fade and, in their attempts to reconstruct the details of what happened, they may have blended the fake psychic’s suggestions in with their blurred memory of the original event in such a way that they now recalled stationary objects as moving. Finally, it is possible that at the time the participants completed the recall questionnaire, they did not actually believe that the stationary objects had moved at all, but simply reported that they did, perhaps believing that this would please the investigators.

The results of the current study can perhaps cast some light upon these competing explanations. We begin by acknowledging that self-report data alone can never definitively distinguish between perceptual effects and memory effects. Even if we ask participants to tell us what they are perceiving as events unfold before them, there will always be a slight delay, perhaps only a fraction of a second, between the perception of the events and the subsequent report. Thus it is always possible to argue that the perception of the events was fundamentally veridical but the memory of the event was somehow distorted. In fact, however, the general position of modern cognitive psychology is that perception and memory are constructive processes and that both will be influenced by bottom-up influences (i.e., raw sensory input) and top-down influences (e.g., beliefs, knowledge, expectations). Thus perception itself is heavily dependent upon memory. When considering ostensibly paranormal events then, both perception (French, 2001) and memory (French, 2003) are likely to be influenced by a variety of top-down influences and thus both perception and memory are likely to be influenced by verbal suggestions that alter expectations.

In considering the experimental set-up used by Wiseman and Greening (2005), it appears that participants completed the FRQ immediately after viewing the video, thus minimizing the possibility that the effect is due to the type of blending of a blurred memory of an essentially accurate perception with the memory of the suggestion, as described above. Although a direct effect upon the actual perception of the event is entirely consistent with Wiseman and Greening’s (2005) data, the possibility of an explanation in terms of demand characteristics remains.

The data from the current study demonstrate unequivocally that social influence provided *after* the video had been viewed was sufficient to alter witnesses’ reports of what they saw. Fully 40% of the participants in the positive social influence condition reported that the key continued to bend even in the absence of a verbal suggestion to that effect from the fake psychic. Furthermore, of those participants who did receive the verbal suggestion, the percentage of participants reporting that the key continued to bend was markedly affected by the reports of the stooge co-witness. Such effects are only explicable as either memory effects or in terms of demand characteristics.

We do not feel that demand characteristics provide a parsimonious explanation of our findings when the responses to item FRQ3b are considered. Data relating to the confidence expressed in the memory report indicate that in both the no social influence and the positive social influence conditions, participants erroneously reporting that the key continued to bend expressed higher levels of confidence than those who did not report that the key continued to bend, thus replicating Wiseman and Greening’s (2005) Experiment 2. In both cases, confidence levels were extremely high (>6 on a 7-point scale). This clearly indicates that expressions of confidence in the accuracy of reports of OPEs should not be taken as any kind of indication of reliability. The lowest confidence ratings in the experiment came from those participants in the negative social influence condition who reported that the key continued to bend and those in the positive social influence condition who reported that it did not. It seems likely that the former group really did perceive the key as continuing to bend and were prepared to stick to that view despite a forceful stooge arguing that it did not. The latter group, on the other hand, did not report that the key continued to bend but their confidence in that view was clearly shaken by a forceful stooge arguing that it had. In both cases, the responses of participants seem to be more in line with participants trying their best to give honest accounts of what they saw rather than behaving in accordance with demand characteristics.

Finally, there is another level at which the influence of beliefs comes into play. The overall interpretation of the demonstration as evidence for the paranormal was, as one might expect, strongly related to paranormal belief. Considering first the believers, it is worth noting that the vast majority did not consider that the demonstration involved paranormal forces—even if they reported that the key continued to bend. Even so, a much higher proportion of believers than disbelievers reported that they had witnessed paranormal forces in action (around 40% of those who reported continued bending of the key). The disbelievers, on the other hand, were much less likely to report that the key continued to bend and, even if they thought it did, they were much less likely to opt for a paranormal explanation. Across the experiment as a whole, of 49 participants who reported that the key continued to bend, only 14 thought that the demonstration involved paranormal forces. The others, presumably, thought that it was some kind of trick, a tendency found much more strongly amongst the disbelievers than amongst the believers. This is not unreasonable, given that such an effect could have been produced by either special effects or by the use of a trick key. It is even possible that some participants realized it was a simple effect of suggestion but were honest enough to admit that it had worked on them. It would be of interest in future studies to ask such participants directly for their explanation of the effect. It would also be of interest in future investigations to include conditions in which the key really does appear to bend to investigate whether disbelievers are prone to deny such events.

It should be noted that one difference between the two social influence conditions and the no social influence condition in the current study was that the former involved discussion of what had been witnessed whereas the latter did not. It is therefore possible that this might have influenced the results in some way, e.g., in terms of differential delay in recall, differences in the number of retrieval attempts, etc. We do not feel this was a major methodological problem with our study as these factors were matched across the positive and negative social influence conditions and thus the impact of different types of social influence are clearly demonstrated by our results. However, we would recommend that similar studies in future replace our current no social influence condition with one that does involve discussion with the stooge participant and the use of the short (4-question) questionnaire ostensibly to facilitate the discussion. The difference would be that the stooge would be presented as someone who has not themselves seen the video clip and ostensibly is simply acting as a facilitator.

### Conflict of Interest Statement

The Review Editor Fiona Gabbert declares that, despite being affiliated with the same institution as authors Krissy Wilson and Christopher French, the review process was handled objectively and no conflict of interest exists. The authors declare that the research was conducted in the absence of any commercial or financial relationships that could be construed as a potential conflict of interest.
